# Oral cancer knowledge and screening behavior among smokers and non-smokers in rural communities

**DOI:** 10.1186/s12885-021-08198-5

**Published:** 2021-04-20

**Authors:** Tzu-Jung Wong, Qian Li, Virginia Dodd, Wei Wang, Jiang Bian, Yi Guo

**Affiliations:** 1 National Dental Research Institute Singapore, National Dental Centre Singapore, Singapore, Singapore; 2 Department of Health Outcomes and Biomedical Informatics, College of Medicine, University of Florida, PO Box 100177, 2004 Mowry Road, Suite 2251, Gainesville, FL 32610-0177 USA; 3 Department of Community Dentistry and Behavioral Science, College of Dentistry, University of Florida, Gainesville, Florida USA; 4 Department of Pharmaceutical Outcomes and Policy, College of Pharmacy, University of Florida, Gainesville, Florida USA; 5 Cancer Informatics Shared Resources, University of Florida Health Cancer Center, Gainesville, Florida USA

**Keywords:** Smoking, Oral and pharyngeal cancer, Health disparities, Rural health, Screening

## Abstract

**Background:**

Research suggests having an oral and pharyngeal cancer (OPC) examination for early diagnosis can increase survival rate. However, the OPC screening rate is low in certain populations. To improve OPC screening rate, this study identified factors that are associated with having an OPC examination.

**Methods:**

Participants with landlines and aged 25 years and older were recruited from six northern Florida counties. Bivariate and logistic regressions were used to predict the outcome of whether the participants had ever had an OPC examination as well as whether participants had ever heard of an OPC examination.

**Results:**

Of 2260 participants with a mean age of 55.9 ± 15.0 years, the majority of participants never smoked (53.4%), self-identified as Whites (70.6%), and had some college or 2-year degree education (30.3%). Smokers were significantly less likely to have ever heard of an OPC examination than those who never smoked. Significant interaction between smoking status and race, and smoking status and social support interaction were found. Whites who never smoked were more likely to have had an OPC examination than non-Whites who never smoked. Former and current smokers with greater social support were more likely to have had an OPC examination than those with lower social support.

**Conclusion:**

The findings from this study inform the need to enhance the awareness of having an OPC examination among smokers and to reduce barriers for racial minority populations to receive an OPC examination. Future research is warranted to develop interventions to target certain populations to improve the rate of OPC examination.

## Introduction

Oral and pharyngeal cancer (OPC) is a deadly disease and treatments are often disfiguring. According to data from the American Cancer Society, nearly 52,000 people were diagnosed with OPC and approximately 10,000 people died from OPC in 2018 in the United States [1]. On the other hand, OPC is highly preventable and early diagnosis can increase chances of survival [2]. Having an OPC examination early is key to detecting OPC at an earlier stage and preventing mortality [3, 4]. Nonetheless, OPC screening rates are low, especially in certain population subgroups, such as males aged between 45 and 64 years old, African Americans, and people with low socioeconomic status [5, 6]. To increase OPC examination rates and survival, it is important to understand what demographic and psychosocial factors are associated with having an OPC examination.

People who engage in risky behaviors such as excess consumption of alcohol and cigarette smoking and who have the presence of human papillomavirus type 16 (HPV-16) infection have been found to be at a higher risk of getting OPC and have a lower OPC survival rate [7–12]. Worldwide, cigarette smoking causes 20 to 30% of oral cancer [13]. Although the overall smoking rate among adults has steadily decreased in the past decades, 14% of adults are smokers in the United States according to 2017 data [14]. Studies have reported an associative relationship between the use of all forms of tobacco products, including direct and indirect use, and OPC [7, 15–18]. Smokers are at a higher risk of death caused by OPC than non-smokers, regardless of gender [7, 19]. According to data from the Behavioral Risk Factor Surveillance System although smokers are more likely to have oral health problems, their dental visit rates are lower compared to non-smokers, regardless of socioeconomic status and other health-related characteristics [20–25]. Dental visit rates are even lower among long-term and heavy smokers [20, 26]. Current smokers aged 40 years or older are at the highest risk of OPC, but are less likely to have an OPC examination than former smokers [5, 13]. The “inverse screening” suggests that people who engage in risky behaviors, such as heavy use of alcohol and tobacco, are less likely to take risk aversion behaviors such as dental attendance and cancer screening [12, 27, 28].

In addition to cigarette smoking, factors such as age, gender, socioeconomic status, race/ethnicity, and health behaviors are associated with OPC examination. People who are older, male, Black, heavy tobacco users, heavy alcohol users, and have low socioeconomic status and poor diet are at a higher risk of developing oral cancer [7, 15, 29, 30]. The results of a survey conducted in 2008 showed that 29.4% of people aged 18 years or older reported ever having an OPC examination in the United States [31]. People aged 18 to 39 years are less likely to have ever had an OPC examination than those aged 40 years or older [31]. Although OPC mortality among Blacks was found to be approximately twice as high as among Whites, the OPC examination rate among Blacks is lower than Whites [2, 32, 33]. Blacks are also at a higher rate of diagnosis with a later stage of OPC than Whites because of delays in OPC screening [34]. However, people who are at a higher risk of OPC are less likely to have regular dental examinations [35]. Barriers to oral cancer screening include lack of routine dental visits, insufficient patient-provider communication due to lack of knowledge, low social attention concerning OPC and risk factors, lack of resources to get an OPC examination, and fear/defensive avoidance [23, 36–43].

Prior research has demonstrated that smokers are at a higher risk of OPC and barriers to having an OPC examination in smokers have been identified. However, there is a lack of studies considering psychosocial factors such as perceived social support, as well as the interactions between demographic and psychosocial factors with smoking status when predicting whether a person has ever had an OPC examination. These interactions may play an important role in decision making related to having OPC screening, but research in this area is critically lacking in Florida. In this study, using a large sample from rural communities in Florida, we aimed to extend prior research by: (1) studying what factors predict the outcome of whether the participants had ever had an OPC examination; and (2) examining what factors predict whether participants had ever heard of an OPC examination. We hypothesized that we would be able to replicate the previous findings that people who had ever heard of an OPC examination were more likely to have had an OPC examination. Next, because evidence suggests that smokers are less likely to have had an OPC screening than non-smokers, we hypothesized that smoking status would be partially responsible for whether the participants had ever heard of OPC screening. In addition, we included psychosocial factors such as social support, comorbid conditions, perceived concerns of getting an OPC screening, and demographics as control variables in our analysis.

## Methods

### Study design

The participants were recruited using the computer-assisted telephone interviews (CATI) technique from April 2010 to February 2011 in six rural, northern Florida counties: Alachua, Bradford, Columbia, Gadsden, Jefferson, Leon, and Union. To ensure Blacks were adequately represented in our sample, we identified the rural census tracts that had populations greater than 30% Black within these counties and oversampled Blacks. The study was executed by professional interviewers at the University of Florida Bureau of Economic and Business Research Survey Center. Households with landlines were selected and only one participant was chosen from each household. The household was considered ‘uncontactable’ if no one had answered to any of 10 telephone calls. The oldest male within the household was prioritized during selection to maximize the participation of older men and balance representation of gender. A total of 16,000 telephone numbers was dialed, resulting in 2605 people aged 25 years and older who lived in the six rural counties participating in the current study.

### Variables of interest

#### OPC examination

The main outcome variable of interest was whether the participants ever had an OPC examination. We asked the participants “Have you ever HAD a mouth or throat cancer examination?” Participants who responded *No* were coded as 0 and those who responded *Yes* were coded as 1.

#### Ever heard of an OPC examination

The second main outcome variable of interest was whether the participants ever heard of an OPC examination. We asked the participants “Have you ever HEARD of an examination for mouth or throat cancer examination?” Participants responding *No* were coded as 0 while those responding *Yes* were coded as 1.

#### Smoking status

We measured smoking status by asking two questions: “In your entire life, have you smoked at least 100 cigarettes, about 5 packs?” and “Do you now smoke cigarettes every day, some days, or not at all?” We categorized the participants into three groups: current smokers, former smokers, and never smoked (reference group). Participants who had smoked at least 100 cigarettes in their lifetime and reported smoking “everyday” or “some days” at the time of the survey were categorized as current smokers. Those who reported they had smoked at least 100 cigarettes over the course of their lifetime and identified themselves as not smoking at all currently were categorized as former smokers. People who never smoked refer to those who had never smoked at least 100 cigarettes in their lifetime and reported they did not smoke at all at the time of the survey.

#### Demographic and psychosocial variables

We collected sociodemographic variables, including age (continuous, range = 25–99 years), gender (dichotomous, men or women; women were the reference group), race (dichotomous, white or black; Blacks were the reference group), education (categorical, range = 1–6, higher numbers indicated higher educational attainment), and a financial security score. The financial security score was computed as the weighted average of the following two items: (1) “Which of these statements best describes your present financial status?” and (2) “If you were faced with an unexpected $500 medical bill that was not covered by insurance, how would you best describe your situation?” Possible scores for the first item ranged from 1 (I really cannot make ends meet) to 4 (money is not a problem, I can buy about whatever I want) and the possible scores for the second item ranged from 1 to 3, with higher scores reflecting being more able to comfortably pay the bill. The financial security score ranged from − 1 to 1; a higher score indicated a higher level of financial security. The question, “How concerned are you about getting mouth or throat cancer in the future?” was conducted to assess perceived concern of the participants. The item responses ranged from 1 (definitely not concerned) to 4 (very concerned) but were categorized into three groups (1 = not concerned, 2 = little concern, and 3 and 4 = very concerned). We measured health literacy by asking, “How often do you have a problem understanding the written materials about your medical condition?” and “How often do you have a problem understanding what is told to you about your medical condition?” (1 = rarely or none of the time, 4 = all of the time). Questions on chronic conditions were drawn from the Seattle Index of Comorbidity. We asked the participants, “Has a doctor or nurse has ever told you that you have any of the following problems?: cancer (excluding skin cancer), chronic lung disease or emphysema, asthma or bronchitis, congestive heart failure, diabetes, heart attack, pneumonia, stroke, arthritis, and chronic back pain.” Chronic conditions, ranging from one to ten, indicated the total number of coexisting chronic conditions. We categorized the participants into three groups: no chronic conditions, one chronic condition, and two or more than two chronic conditions. Social support was accessed using five items from a modified version of the medical Outcomes Social Support (MOSS) survey. The participants were asked to report how often, if needed, each of the following kinds of support was available to them: “someone to help with daily chores if you are sick,” “someone to turn to for suggestions about how to deal with a personal problem,” “someone to do something enjoyable with,” “someone to love and make you feel wanted,” and “someone to take you to the doctor if you needed it” (1 = none of the time, 5 = all of the time). We computed the average score of answered questions as the social support score. The score ranged from 0 to 4, a higher score indicated perceived greater social support [24, 44, 45].

### Data analysis

Differences in demographic and psychosocial variables by smoking status were tested using survey-sample-weighted t tests (for continuous variables) or chi-squared tests (for categorical variables). We built survey-sample-weighted multiple logistic regression models to assess the association of the two outcome variables (whether the participants had ever had an OPC examination and whether participants had ever heard of an OPC examination and predictor variables including age, education, and financial security, gender, race, ever heard of an oral concern, health literacy, perceived concern, chronic conditions, social support, smoking status, and their interactions with smoking status. We used the survey procedure PROC SURVEYLOGISTIC of SAS version 9.4 (SAS Institute, Cary, NC) for the analysis.

## Results

### Participants’ characteristics

We summarized characteristics of the participants stratified by smoking status in Table 1. The mean age of the 2260 participants in the study was 55.9 ± 15.0 years. The majority of participants never smoked (*n* = 1206, 53.4%), self-identified as Whites (*n* = 1595, 70.6%), and had some college or 2-year degree (*n* = 684, 30.3%). There was a significant difference in the distribution of gender, race, education, age, and financial security score across people who never smoked, were former smokers, and current smokers (*p* < .0001). In addition, there were significant differences between groups with respect to ever having an OPC examination, ever hearing of an OPC examination, health literacy, concern, chronic conditions, social support, and depression (*p* < .001).

**Table 1:** Participant Characteristics by Smoking Status

Variable	Never Smoker (***n*** = 1206), Mean ± SD or No. (%)	Former Smoker (***n*** = 674), Mean ± SD or No. (%)	Current Smokers (***n*** = 380), Mean ± SD or No. (%)	Total (***N*** = 2260), Mean ± SD or No. (%)	***p***
**Age (years)**	55.0 ± 15.0	60.1 ± 13.4	51.1 ± 13.5	55.9 ± 14.6	***< .0001***
**Gender**					
Female	744 (61.7)	318 (47.2)	198 (52.1)	1260 (55.8)	***< .0001***
Male	462 (38.3)	356 (52.8)	182 (47.9)	1000 (44.2)	
**Race**					
Black	441 (36.6)	117 (17.4)	107 (28.2)	665 (29.4)	***< .0001***
White	765 (63.4)	557 (82.6)	273 (71.8)	1595 (70.6)	
**Financial security (Range − 1 ~ 1)**	0.2 ± 0.6	0.2 ± 0.6	−0.2 ± 0.6	0.1 ± 0.6	***< .0001***
**Education**					
8th grade or less	18 (1.5)	13 (1.9)	12 (3.2)	43 (1.9)	***< .0001***
HS (Not graduated)	62 (5.1)	40 (5.9)	53 (13.9)	155 (6.9)	
HS/GED (Graduated)	303 (25.1)	182 (27.0)	128 (33.7)	613 (27.1)	
Some college / 2-year degree	337 (27.9)	213 (31.6)	134 (35.3)	684 (30.3)	
4-year college graduate	217 (18.0)	121 (18.0)	29 (7.6)	367 (16.2)	
More than 4-year college degree	269 (22.3)	105 (15.6)	24 (6.3)	398 (17.6)	
**Depression (CES-D)**					
No	956 (79.3)	532 (78.9)	236 (62.1)	1724 (76.3)	***< .0001***
Yes	250 (20.7)	142 (21.1)	144 (37.9)	536 (23.7)	
**Chronic conditions**					
0	479 (39.7)	160 (23.7)	117 (30.8)	756 (33.5)	***< .0001***
1	287 (23.8)	163 (24.2)	82 (21.6)	532 (23.5)	
≥ 2	440 (36.5)	351 (52.1)	181 (47.6)	972 (43.0)	
**Ever heard of OPC exam**					
No	635 (52.7)	332 (49.3)	236 (62.1)	1203 (53.2)	***.0002***
Yes	571 (47.3)	342 (50.7)	144 (37.9)	1057 (46.8)	
**Concern**					
Not concerned	548 (45.4)	254 (37.7)	68 (17.9)	870 (38.5)	***< .0001***
Little concern	411 (34.1)	292 (43.3)	157 (41.3)	860 (38.1)	
Very concerned	247 (20.5)	128 (19.0)	155 (40.8)	530 (23.5)	
**Ever had OPC exam**					
No	636 (52.7)	322 (47.8)	249 (65.5)	1207 (54.3)	***< .0001***
Yes	570 (47.3)	352 (52.2)	131 (34.5)	1053 (46.6)	
**MOSS (Range 0 ~ 4)**	3.0 ± 0.9	3.0 ± 1.0	2.8 ± 1.0	2.9 ± 1.0	***.0004***
**Health literacy (Range 0 ~ 3)**	2.4 ± 0.7	2.4 ± 0.7	2.2 ± 0.8	2.4 ± 0.7	***< .0001***

Note: *HS* High School, *GED* General Equivalency Diploma, *CES-D* Center for Epidemiologic Studies Depression, *MOSS* Medical Outcomes Social Support

All percentages are survey sampling weighted. The *p*-values smaller than .05 are bolded

### Predicting OPC examination

We summarized results from the logistic regression analysis predicting whether participants ever had an OPC examination in Table 2. In the analysis, we included age, education, financial security, gender, race, ever heard of an oral concern, health literacy, perceived concern, chronic conditions, social support, smoking status, and the interactions of these variables with smoking status as predictors. The results showed that older people were 1.09 times more likely than younger people to have had an OPC examination (odds ratio [OR] = 1.09; 95% confidence interval [CI] = 1.02–1.17; *p* = .018). Participants with a higher education level, a higher financial security score, and two or more than two chronic conditions were more likely to have had an OPC examination than those with a lower education level, a lower financial security score, and no chronic conditions (OR = 1.20; 95% CI = 1.11–1.31; *p* < .0001; OR = 1.68; 95% CI = 1.39–2.01; *p* < .0001; OR = 1.41; 95% CI = 1.11–1.79; *p* = .0046). In addition, people who were very concerned about getting OPC in the future and had ever heard of an oral examination were more likely to have had an OPC examination than those who were not concerned about getting OPC in the future and those who had never heard of an OPC examination (OR = 1.31; 95% CI = 1.01–1.70; *p* = .0456; OR = 3.73; 95% CI = 3.11–4.49; *p* < .0001). In predicting the outcome of “Ever had OPC examination”, former smokers were more likely to have had an OPC exam compared to never smokers (OR = 1.75; 95% CI = 1.11–2.76) or current smokers (OR = 2.27; 95% CI = 1.23–4.21), but only among Blacks. Smoking status is not predictive of OPC exam among Whites. The analysis revealed a significant smoking status by race interaction and smoking status by social support interaction. Whites who never smoked were 1.5 times more likely to have an OPC examination than non-Whites who never smoked (OR = 1.52; 95% CI = 1.16–2.00; *p* = .00026). Furthermore, former and current smokers with greater social support were more likely to have had an OPC examination than those with lower social support (OR = 1.22; 95% CI = 1.02–1.45; *p* = .0277; OR = 0.78; 95% CI = 0.62–0.98; *p* = 0.0339).

**Table 2:** Odds Ratios (95% CI) in the Logistic Regression Model for Predicting the Outcome Variables

Variables	Ever had OPC examination	Ever heard of OPC examination
***Main predictors***		
**Ever heard of OPC examination**	3.733*** (3.105–4.487)	–
**Smoking Status**		
Never smoker (Reference)		
Former smoker	–	1.092 (0.893–1.335)
Current smoker	–	0.774* (0.599–0.999)
**Race by smoking status**		
White vs Other at never smoker	1.520** (1.158–1.996)	–
White vs Other at former smoker	0.794 (0.507–1.243)	–
White vs Other at current smoker	1.575 (0.913–2.717)	–
**Social support by smoking status**		
Social support at never smoker	0.973 (0.848–1.116)	–
Social support at former smoker	1.217* (1.022–1.450)	–
Social support at current smoker	0.782* (0.623–0.982)	–
***Control variables***		
**Age (10-years)**	1.090* (1.015–1.171)	0.984 (0.922–1.052)
**Gender**	1.003 (0.831–1.211)	1.045 (0.879–1.242)
**Race**	–	1.383** (1.129–1.693)
**Education**	1.201*** (1.105–1.307)	1.061 (0.992–1.135)
**Financial security**	1.676*** (1.394–2.014)	1.438*** (1.216–1.701)
**Health literacy**	0.996 (0.860–1.153)	1.229**(1.074–1.407)
**MOSS**	–	0.986 (0.899–1.081)
**Depression (CES-D)**	0.826 (0.650–1.049)	0.828 (0.665–1.031)
**Chronic conditions**		
No chronic condition (Reference)		
1 chronic condition	1.089 (0.849–1.398)	1.043 (0.830–1.312)
2 or more than 2 chronic conditions	1.411** (1.112–1.789)	1.141 (0.917–1.419)
**Perceived concern**		
Not concerned (Reference)		
Little concern	1.132 (0.916–1.400)	1.081 (0.889–1.313)
Very concerned	1.307 (1.006–1.697)	1.122 (0.884–1.425)

Note: *CES-D* Center for Epidemiologic Studies Depression, *CI* Confidence Interval, MOSS Medical Outcomes Social Support, *OPC* Oral and Pharyngeal Cancer. *p < .05; **p < .01; ***p < .001

We summarized results from the logistic regression analysis predicting whether participants had ever heard of an OPC examination in Table 2. In the analysis, we included demographic variables, health literacy, perceived concern, chronic conditions, and smoking status as predictors. The results showed that Whites were 1.38 times more likely than non-Whites to have ever heard of an OPC examination (OR = 1.38; 95% CI = 1.13–1.69; *p* = .0017). Participants with a higher financial security score and a higher health literacy level were more likely to have ever heard of an OPC examination than those with a lower financial security score and a lower health literacy level (OR = 1.44; 95% CI = 1.22–1.70; *p* < .0001; OR = 1.23; 95% CI = 1.07–1.41; *p* = .0027). In addition, the analysis indicated that smokers were significantly less likely to have ever heard of an OPC examination than those who never smoked (OR = 0.77; 95% CI = 0.60–1.00; *p* = .049).

## Discussion

### Principal findings

This research aimed at exploring whether smoking status predicts the likelihood of ever having heard of an OPC examination as well as the likelihood of ever having had an OPC examination, taking into account interactions between smoking and demographic and psychosocial variables. We found that smokers were less likely to have heard of an OPC examination than non-smokers. In addition, among non-smokers, Whites were more likely to have an OPC examination than non-Whites. Former and current smokers with greater social support were more likely to have an OPC examination than those with lower social support. This is the first study, to our knowledge, that analyzed a large sample from rural communities in Florida to discover the factors associated with knowledge of OPC examinations, as well as factors associated with having ever had an OPC examination among smokers and non-smokers.

Our results showed that people who never smoked were more likely to have ever heard of an OPC examination than smokers. Furthermore, among Blacks, never smokers were less likely to have had an OPC exam than former smokers. One possible explanation of the lower rate of ever having heard of an OPC examination among smokers is that smokers are less likely to have regular dental visits [12, 20–25]. Previous studies have identified that dentists play an important role in informing patients and the public about the signs, symptoms, and knowledge about the etiology of OPC, and disseminating knowledge about the need to have an OPC examination [24, 46]. As a result of a lack of dental visits, smokers have been shown to be more likely to have a poorer oral health status than non-smokers, especially among low-income populations [20, 23, 24, 27, 42, 43]. In our survey, participants were asked to report whether they have ever heard of an OPC examination first then were asked to report whether they have ever had an OPC examination with a description of the exam procedure (e.g., dentist checking the inside of your mouth). It is possible that the former smokers may have not heard of an OPC examination but have had received an OPC examination during their dental visits provided by their health providers as former smokers are at a higher risk of OPC than never smokers [47]. As greater screening intention is associated with the recommendation provided by their healthcare providers; without regular dental visits, smokers may not receive any recommendations from their dentists and hence are less likely to have heard of an OPC screening [46]. Further research is warranted to understand the reasons for not having regular dental visits among smokers and interventions to motivate smokers to have regular dental visits should be developed.

The results of this study also showed that racial disparities had an impact on the relationship between smoking status and OPC examination. Whites who never smoked had a higher rate of OPC examination than non-Whites who never smoked. The lack of knowledge regarding the importance of having an OPC examination and lack of concern about getting OPC leads to never having had an OPC examination. Detecting OPC at an early stage may decrease its mortality rate. Research has identified that barriers to screening for OPC among non-Whites include lack of resources, defensive avoidance, as well as lack of knowledge and social attention [46]. Research has been focusing on delivering messages to increase the awareness of OPC through the media [2, 48–50]. People who have been exposed to the messages have increased concern about getting OPC, especially Blacks, and were more likely to have received their first OPC examination [2]. It is important to develop such kinds of interventions that target Black smokers and examine the long-term effects of the interventions.

Finally, our results indicated that former and current smokers with greater social support were more likely to have an OPC examination than former and current smokers with lower social support. This finding is consistent with previous studies that demonstrated personal coping resources, including social support, is one of the key factors that influence the likelihood of getting an OPC examination [51–54]. People with more coping resources are more receptive to potential undesirable health information than those with less coping resources [55].

### Limitations

Results from this study should be interpreted within its limitations. This study may have sampling bias because the sample was drawn from people with landlines. However, our target population was older adults, among whom landlines are popular [56]. Another limitation is that this was a survey study and hence was subject to memory and recall bias. Some participants may not be able to recall whether they have received an OPC examination. The prevalence of people who had ever received ana OPC examination may be overestimated as a previous study showed that minorities often over-report having had an examination [57, 58]. Additionally, we only had data on cigarette smoking. Future studies should access the use of other tobacco products, such as chewing tobacco and cigars, which could also increase the risk of dental caries and oral cancer. Lastly, the current study only recruited the participants in rural north Florida and accessing whether the participants have ever received an OPC examination in the dental settings, the lack of more sampling and clinical sites limits the generalization of the findings.

## Conclusions

This study provides data on whether people in a large population of rural communities in Florida have ever heard of an OPC examination and have ever had an OPC examination. Our results highlight the importance of advancing interventions targeting smokers to increase the awareness of having an OPC examination. Policies to reduce racial disparities to reduce the barriers to having an OPC examination should be established. In addition, future research is needed to develop interventions to improve social support for former and current smokers to increase the rate of OPC examination. While the findings from this study suggest that whether the respondents had ever heard of an OPC examination may be an important factor in changing the behavior of having an OPC examination, we cannot demonstrate causality. Future research is needed to understand the causal relationship and to develop interventions to improve the rate of OPC examination.

## Data Availability

The datasets supporting the conclusions of this article contain protected health information (PHI) such as names and addresses, so they are not available for public use. Please contact the corresponding author for further details.

## Abbreviations

CES-D: Center for Epidemiologic Studies Depression
CI: Confidence interval
HPV-16: Human papillomavirus type 16
MOSS: Medical Outcomes Social Support
OPC: Oral and pharyngeal cancer
OR: Odds ratio
